# Use of an innovative electronic communications platform (912Rwanda) to improve prehospital transport of injured people in Rwanda: protocol for a type 2 hybrid effectiveness-implementation interrupted time series study

**DOI:** 10.1136/bmjopen-2025-100826

**Published:** 2025-08-13

**Authors:** Rwanda912 RIGHT Group, Laura Quinn

**Affiliations:** 1 Department of Applied Health Sciences, Birmingham, UK

**Keywords:** ACCIDENT & EMERGENCY MEDICINE, Wounds and Injuries, Implementation Science

## Abstract

**Abstract:**

**Introduction:**

Injury is a major cause of death in Rwanda, with many deaths occurring before hospital admission. Timely transport of injured patients to appropriate hospitals is crucial, ideally within an hour for severely injured patients. However, delays in reaching treatment facilities are common, with ambulance services using inefficient mobile phone communication. This project aims to evaluate the effectiveness and implementation of an innovative electronic communication platform (912Rwanda).

**Methods and analysis:**

The study will be conducted through the public ambulance service, Service d’Aide Médicale d’Urgence (SAMU), and receiving health facilities in Kigali city and Musanze district in Rwanda. The 912Rwanda intervention will be rolled out in the two locations at different times. The primary effectiveness outcome is the time from ambulance deployment to patient arrival at the health facility. Secondary effectiveness outcomes include disaggregated times of the primary outcome and clinical outcomes, such as length of stay and requirement for intensive care. These outcomes will be evaluated using an interrupted time series analysis, accounting for non-homogeneous variances, auto-regressive errors and non-linear trends where appropriate. Implementation outcomes will be evaluated using the Reach, Effectiveness, Adoption, Implementation, Maintenance (RE-AIM) Qualitative Evaluation for Systematic Translation (QuEST) framework. Cost-effectiveness will be evaluated using a cost-consequence analysis with consequences as determined by the interrupted time series analysis.

**Ethics and dissemination:**

Ethical approval was obtained from the Rwanda National Research Ethics Committee (Ref No: 99/RNEC/2023). Dissemination will occur through open-access peer-reviewed publications, relevant national and international conferences.

**Trial registration number:**

ISRCTN97674565.

**STRENGTHS AND LIMITATIONS OF THIS STUDY**

- The use of an interrupted time series approach provides a robust quasi-experimental approach for evaluating intervention’s effectiveness when randomisation is not feasible.
- Implementation will be assessed using the Reach Effectiveness Adoption Implementation Maintenance (RE-AIM) Qualitative and Evaluation Study of Translation (QuEST) framework, which allows for a comprehensive understanding through both quantitative and qualitative outcomes.
- The cost-consequence analysis enables decision-makers to consider a range of outcomes that may be relevant to their decision-making context and to apply their own weights to these alternative outcomes, considering trade-offs between outcomes.
- Generalisability of findings may be limited due to variations in systems and resource availability across low- and middle-income country contexts.
- The rapidly evolving nature of software development and technologies may lead to research findings becoming outdated quickly, making it essential to stay updated with the latest developments.

## Introduction

 Injuries have a profoundly negative effect on both individuals and society.^1^ They cause about 4.4 million deaths globally, with tens of millions more suffering from non-fatal injuries each year.^2^ Adults of working age are primarily affected by injuries, which results in severe physical impairment, long-term disability, and psychological and economic suffering.^3^

Injuries disproportionately affect low- and middle-income countries (LMICs), where over 90% of injury-related deaths occur.^4^ In Rwanda, injuries are estimated to result in about 10% of deaths, with the majority occurring in the prehospital setting.^5^ A pillar of a good trauma service is getting injured patients to the right hospital at the right time. For severely injured patients, this should ideally be within 1–2 hours.^6 7^ Longer times are associated with increased patient mortality.^8^

As in many other LMICs, there are substantial delays in reaching hospital for injured individuals in Rwanda.^5^ Around half of injury deaths in Rwanda occur prehospital and the other half within the first 24 hours of admission.^9^ While some deaths and disability after injuries are inevitable, it is estimated that many deaths after injuries could be avoided if timely access to quality healthcare was available.^9^ In Rwanda, as in other LMICs, many avoidable deaths are due to delays in reaching the right facility in a timely manner.^10 11^

In 2007, the Rwandan Ministry of Health created a public ambulance service called the Service d’Aide Médicale d’Urgence (SAMU). This service was created to provide timely prehospital care to strengthen the health system.^12^ SAMU, originally established as an ambulance service based in the capital, Kigali, has grown to become a nationwide service covered by community-based health insurance.^13^ More than 300 ambulances are deployed, linked by a national dispatch centre, and a free emergency service number (912).^14^

**Figure 1:**
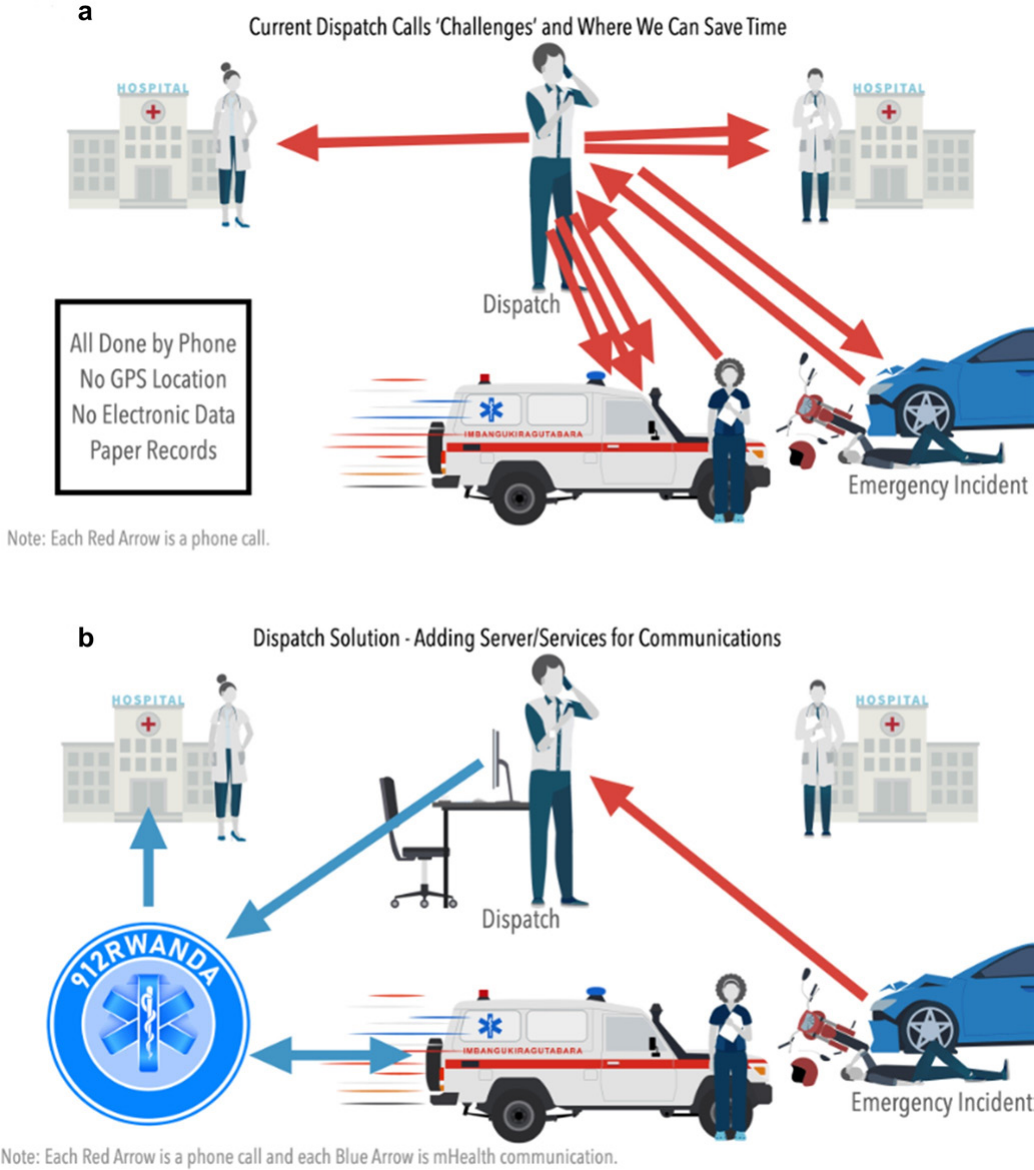
The top figure (part a) represents the current inefficient communication system, which relies on separate mobile phone and very high frequency (VHF) radio communication between the dispatch centre, ambulance crews and health facilities (without global positioning system (GPS) location), resulting in prehospital delays. The bottom figure (part b) represents the proposed 912Rwanda platform, an integrated electronic system designed to connect dispatch, ambulance crews and health facilities to reduce prehospital delays. Image created by coauthor Rob Rickard (Rwanda Build).

Similar to other ambulance services in LMICs, all communications are made by mobile phone and very high frequency (VHF) radio.^15^ Initial explorations found that inefficient mobile phone communication and coordination between ambulance, dispatch and receiving hospitals, and lack of efficient usage of triage data from ambulance staff resulted in an average time to reach hospital of around 1 hour (figure 1, part a). One major consequence of this poor communication is that ambulance crews often lack enough information to find their patients quickly, to identify an appropriate receiving hospital for the patient or to prepare the receiving hospital in advance, wasting valuable time that could be used transporting patients to care. This represents up to 30 min of additional time from the expected journey time from injury location to hospital, with around two-fifths of trips taking longer than 1 hour.^16^ Formative work between industry, academia and policy makers led to the concept of the 912Rwanda electronic communication platform designed by the Rwanda Build Program (RWBuild), a local software company^17^ (figure 1*,* part b). The goal of the 912Rwanda platform is to reduce transport time for injured patients by connecting the patient, hospitals, ambulance crews and dispatch through the platform, reducing need for phone calls. A separate development protocol paper describes the development, training and roll-out of the 912Rwanda platform in detail.^18^ The 912Rwanda platform will be developed in multiple phases. Phase one involves the development and roll-out of software for ambulance dispatch to collect incident details, locate emergencies and enable ambulance staff to receive information. Phase two will focus on the development and integration of the destination decision support algorithm (DDSA) within the 912Rwanda platform, with additional phases launched based on local needs.

### Aim

The overall aim of our study is to evaluate the effectiveness and implementation of an electronic DDSA within the 912Rwanda platform, referred to as the integrated DDSA 912Rwanda platform, designed to improve prehospital transport of injured people in Rwanda. The specific objectives are as follows:

1. To assess the effectiveness of the integrated DDSA 912Rwanda platform in improving prehospital transport of injured people in Rwanda.
2. To evaluate the implementation of the integrated DDSA 912Rwanda platform.
3. To perform a cost-consequence analysis (CCA) of the integrated DDSA 912Rwanda platform compared with current practice.

## Methods and analysis

### Study design

The Rwanda912 project is a hybrid type 2 effectiveness implementation study. In summary, the effectiveness of the integrated DDSA 912Rwanda platform will be evaluated using an interrupted time series design. Implementation will be evaluated using the Reach, Effectiveness, Adoption, Implementation, Maintenance (RE-AIM) Qualitative Evaluation for Systematic Translation (QuEST) framework. Cost-effectiveness of the 912Rwanda platform will be evaluated using a CCA.

### Study setting

The study will be conducted in Kigali city and Musanze district, representing areas with predominantly urban and rural catchments, respectively. In each of these areas, the public ambulance service, SAMU, carries emergency patients to all public and private health facilities. Kigali city is the capital and largest city of Rwanda, located in the nation’s geographical centre. In contrast, Musanze district is located in the Northern Province of Rwanda, making these two areas geographically separate.

In Kigali, at the time of study development, there were 5 district hospitals, 3 referral hospitals and 37 health centres (which receive less urgent cases). In 2021/2022, half of patients were transferred to three hospitals: Kibagabaga District Hospital (21%), Nyarugenge District Hospital (16%) and Centre Hospitalier Universitaire de Kigali (13%). Over 30% of patients were either transferred to health centres or managed on-site for minor cases, with the remaining patients being transferred to other hospitals (online supplemental file 1).

In the district of Musanze, Ruhengeri Level 2 Teaching Hospital (RL2TH) serves as both the referral hospital and the only district hospital. The typically patient journey for emergencies begins at the local health centre, where patients are initially triaged and stabilised. If necessary, patients are referred to RL2TH and occasionally from there to hospitals in Kigali for further treatment. There are currently no means of prioritising patients in health centres for transfer, and 912Rwanda will be used in this case to triage patients from local health centres to RL2TH, and subsequently, to the appropriate hospital in Kigali. In Musanze, there are five available ambulances, with two typically occupied with transferring patients to Kigali and a dedicated team of seven nurses who oversee prehospital care.

### Eligibility criteria

The eligibility criteria for patient inclusion differ between the two study sites. In Kigali, all patients transferred from the incident site to a treatment facility will be included in the analysis. This encompasses transfers to both public and private health facilities within the city. In Musanze district, given that the majority of emergency patients first present to a health centre, and therefore ambulance services to pick patients up from the scene of the emergency are absent, the study will include patients transferred from health centres to the local hospital. However, patients transported from Musanze hospital to a hospital in Kigali will not be counted in the analysis.

### Intervention

The intervention, 912Rwanda, consists of an interface on mobile devices used by ambulances and hospital staff, computer interfaces for dispatch centre staff and a software platform. 912Rwanda will be developed in multiple phases. The focus of this research is on phase two; however, the effect of phase one will also be measured.

Phase one of 912Rwanda involves the development and roll-out of software only in Kigali for ambulance dispatch to collect incident details, use global positioning system (GPS) to locate and navigate to emergencies, and enable ambulance staff to receive information. It also includes an short message service (SMS) tool for communicating incident details to receiving facilities. This phase has been fully implemented in Kigali.

Phase two of 912Rwanda will focus on the development and integration of the DDSA into the software in Kigali and Musanze, the integrated DDSA 912Rwanda platform. In Musanze, a modified version of phase two of 912Rwanda will be implemented based on local needs. The DDSA will use decision algorithms to match the patient to the closest ready facility, accounting for patient medical needs and proximity. The DDSA then alerts the ambulance teams and/or dispatch of the selected facility and the rationale for this. This decision is either approved or overridden. If approved, the ambulance proceeds to the facility. If over-ridden, the ambulance team and/or dispatch (to be decided) manually enters their choice of facility and their rationale for this by means of a pre-populated survey form.

### Patient and public involvement

Extensive stakeholder and community involvement has shaped the development of this research proposal over the 2 years leading to submission. A 2-day workshop with service providers, users and other stakeholders identified improving prehospital transport for patients to appropriate facilities as a key priority. Focus group discussions with patients and community leaders identified barriers to accessing care, highlighting more efficient transport as a priority. Feedback was provided by people with lived experience (eg, patients or family members) of accessing care for injury, which was used to further refine the proposal.

To ensure ongoing community engagement throughout the project, we will establish an Injured Persons Community Group (IPCG) for Kigali and Musanze, with representation of all sections of society in this group (including those hard to reach). IPCG members will contribute to contextualising research tools, interpreting study findings through visual participatory analysis, developing and contributing to dissemination of the project results, and in policy discussions and strategy by empowering IPCG members to be involved in setting local or national agenda for improved injury care.

### Objective 1: effectiveness of intervention

#### Outcomes

The primary effectiveness outcome is the time from ambulance deployment to the patient’s arrival at a hospital (table 1). Secondary outcomes include disaggregated times of the primary outcome and the following clinical outcomes: in-hospital mortality, length of stay and requirement for intensive care.

**Table 1:** Effectiveness outcomes

Outcomes	Description
Primary outcome	Time from ambulance deployment to arrival at facility
Secondary outcomes	In-hospital mortality
	Length of stay
	Requirement for intensive care
	Time from ambulance deployment to arrival at scene
	Time from arrival at scene to leaving scene
	Time from leaving scene until arriving at facility
	Time waiting to be seen after arrival at facility
Exploratory outcome	Time from dispatch call to arrival at facility*

* Only available from phase one implementation onwards.

#### Explanatory variables

The following explanatory variables will be collected: location of injured patients; facility taken to; complaint type; the Triage Early Warning score; sex; age; and presence of community-based health insurance.

#### Intervention timeline

**Figure 2:**
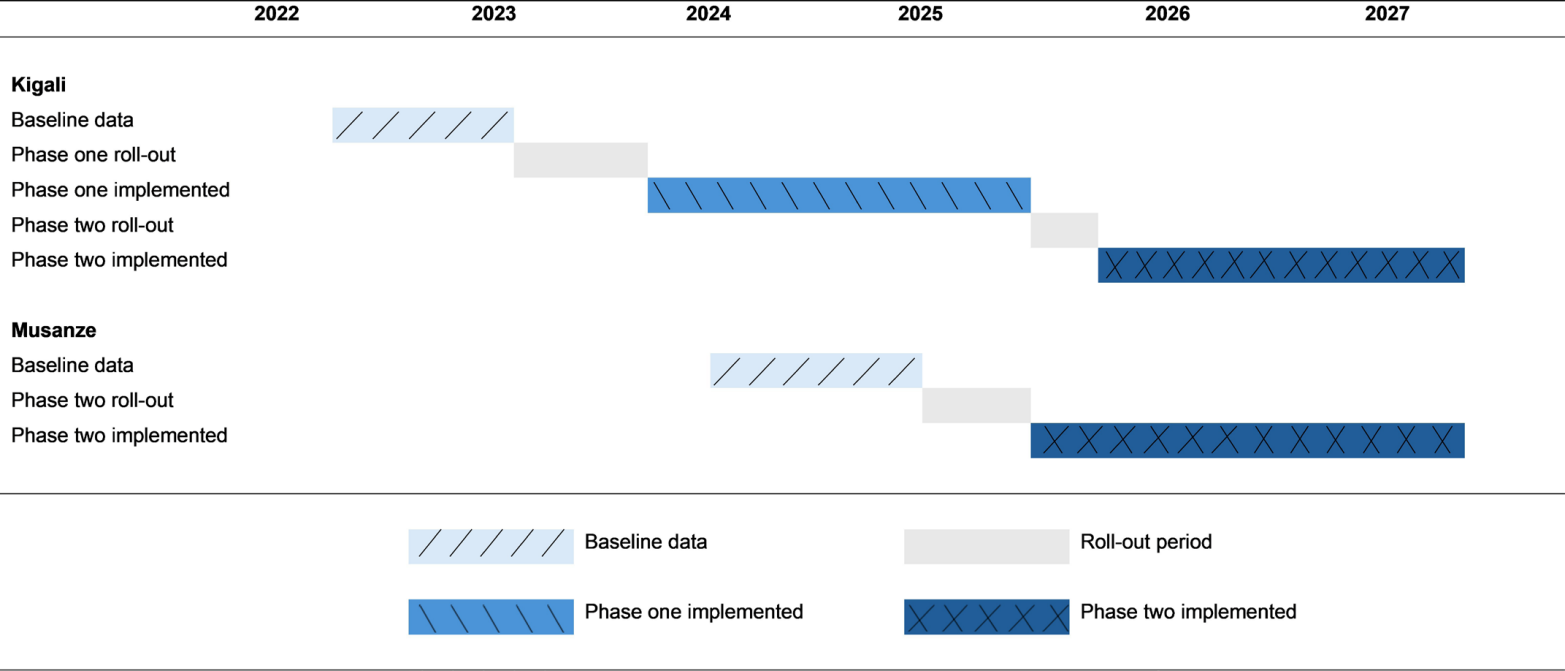
Timeline for roll-out of 912Rwanda in Kigali and Musanze sites. Light blue represents baseline period with no intervention, grey represents roll-out periods, medium blue represents phase one implementation and dark blue represents phase two implementation (which includes phase one implementation).

The roll-out of the 912Rwanda intervention occurs at different times in the two study locations, as shown in figure 2. In summary, we have the following:

For Kigali city:

- Baseline data: July 2022 to February 2023
- Phase one roll-out: March 2023 to November 2023.
- Phase one implemented: December 2023 to July 2025.
- Phase two roll-out: August 2025 to October 2025.
- Phase two implemented: November 2025 to June 2027.

For Musanze district:

- Baseline data: February 2024 to February 2026.
- Phase two roll-out: March 2026 to July 2026.
- Phase two implemented: August 2026 to June 2027.

The timeline shows the staggered implementation of the intervention across the two locations, with Kigali city receiving both phases of the intervention, while Musanze district implements a modified version of phase two only.

#### Data collection

##### Time data

In Kigali, time outcome data for the baseline pre-intervention period will be collected through a time data collection app (TDCA). Time data for phase one and phase two of the intervention will be collected through the 912Rwanda platform. Data will also be collected on paper and inputted electronically as a backup throughout the study period, on which data quality checks will be performed. In Musanze, data for the baseline period will only be collected on paper and inputted electronically, while data for phase two will be collected using the 912Rwanda platform. All electronic time stamps (TDCA and 912Rwanda) are generated automatically from synchronised system clocks to ensure consistency; manual entry of time data is only used as a backup.

##### Clinical and explanatory data

Data for the clinical secondary outcomes and explanatory variables will be collected through existing trauma registries at the referral hospitals in Kigali and Musanze.

### Sample size calculation

For Kigali, which has two phases of the intervention, the sample size is based on the number of expected observations per week (ie, number of ambulance calls) of at least 50. The sample size includes 31 weeks of baseline data, 87 weeks of phase one implementation and 87 weeks of phase two implementation. We have assumed a realistic auto-regressive correlation of order 1 (exponential decay) to summarise the data’s correlation structure. A moderate effect size (standardised effect size of 0.5) is assumed, accounting for the total effect size, which is the sum of the expected level change and trend change in the two phases over the SD. This standardised effect size of 0.5 represents a target effect of half of the SD of the outcome across weekly time periods.

For example, if the average time from ambulance call to facility arrival has a mean of 70 min and SD of 30 (across 108 weeks), this would be equivalent to a total intervention effect of 15 min. A total sample size of 108 observations (weekly means of duration from ambulance deployment to hospital), with an equal number of observations across the three periods, would provide more than 80% power across nearly all auto-regressive correlations (autocorrelation <0.7) at 5% significance. This is a conservative estimate as we have over 87 weekly observations in each post-roll-out period for both phases of the intervention.

For Musanze, which has only one phase of the intervention, we use a similar approach but adjust for the simpler design. The sample size includes 104 weeks of baseline data and 48 weeks of phase two implementation. Assuming the sample weekly rate of ambulance calls, same auto-regressive correlation structure and a moderate effect size (standardised effect size of 0.5), which is the sum of the expected level and trend change in the one phase intervention over the SD, a total sample size of 72 observations would provide more than 80% power across all auto-regressive correlations values of less than 0.6.

### Data analysis

#### Data checking and outlier analysis

Data for the baseline will be verified against manually recorded, electronically inputted records to ensure accuracy and consistency. This process involves matching data based on incident IDs when available. If incident IDs are not available, data will be matched based on deployment time.

For each phase (baseline, phase one and phase two), minimum and maximum thresholds for the primary and secondary outcomes will be established. These thresholds will be determined based on expert opinion. This approach helps in identifying potential outliers in the dataset.

#### Data analysis overview

The primary, secondary and exploratory effectiveness outcomes will be summarised in weekly periods, with separate analyses conducted for Kigali and Musanze sites. Interrupted time series models will be used independently at each site to evaluate the effect of the intervention, thereby providing intervention effects for each location. The specific interrupted time series methods used will follow best practice guidelines from a simulation comparing different statistical methods.^19^

#### What we will estimate (intervention effects to be estimated)

For Kigali, the model will evaluate both the effect of phase one and two of the 912Rwanda intervention. Estimates for the pre-intervention trend, shift changes when each of the phases of the intervention is implemented and post-intervention trends will be reported with 95% CIs. We will also estimate the combined effect of both phases of the intervention by using linear combinations of the coefficients for the shift changes and trend changes associated with each phase. Additionally, we will analyse the exploratory outcome of time from dispatch call to arrival at facility, for phase two of the intervention.

For Musanze, the model will evaluate only the modified phase two of the intervention. Estimates for the pre-intervention trend, shift change when phase two of the intervention is introduced, and the post-intervention trend will be reported with 95% CIs.

#### How we will estimate these effects

An initial Auto-Regressive Integrated Moving-Average (ARIMA) model will be fitted, including a time indicator (weekly summaries), intervention phases indicators (pre-intervention, roll-out, post-intervention) and interactions between time and intervention phases.

Diagnostic checks will be performed, and the model will be updated as needed to account for non-homogeneous variances, auto-regressive errors and non-linear trends. Regression diagnostic plots will be examined to reinforce correct transformations and error structures used.

#### Non-homogeneous variance

Homogeneity of the variance will be examined by plotting the local means of a set of consecutive observations against their SD. A clear relationship between the SD and level of the series would suggest non-homogeneous variances. This will be explored on both original and log scales, and transformations will be considered if necessary.

#### Auto-regressive errors

Autocorrelation function plots (ACF), partial ACF (PACF) and Ljung-Box tests will identify any non-random periodicity. The ACF and PACF plots will be used to determine the type and order of autocorrelation, with the PACF showing autocorrelation adjusting for intervening lags. Existing guidelines will be used to identify the autocorrelation type from these plots, for example, a significant spike at lag 1 only suggests an auto-regressive (AR1) or exponential decay structure.^20^

#### Non-linear trends

To account for any strong non-linear trend intervention effects, a polynomial spline function (eg, cubic spline) will be considered. Recommended approaches will be used to identify the appropriate number of knots. Only statistically significant non-linear terms will be included in the model. However, a cautious approach to modelling non-linear trends will be taken, as splines can be unreliable at time period endpoints, which coincide with the points of inference for interrupted time series. Additionally, including non-linear terms reduces study power and complicates interpretation as there are multiple trend coefficients instead of single pre/post-intervention coefficients.

#### Allowance for confounders

Interrupted time series analysis uses aggregate level data, limiting the possibility of adjusting for individual-level covariates. Nonetheless, the influence of confounders might be important. We will assess if there is evidence of participant’s characteristics (eg, age, sex) over the study duration. Any clear evidence of changes will be an important signal that analysis needs to be adjusted for confounders, or interpretation should account for confounding effects.

If there is evidence of confounding and minimal autocorrelation, we will use ordinary least squares (or logistic regression for binary outcomes) to allow for individual level covariate adjustment. Previous research has shown if autocorrelation is low, then this approach provides optimal effect estimates.^19^

If autocorrelation is identified, adjustment will be made using aggregate covariate measures at each time point.

#### Missing data

Missing data may occur for covariate or outcome data. When using aggregate covariate data, we will explore the robustness of various approaches, using simulation-based methods to account for missing data, as standard multiple imputation methods are not amenable. Missing outcome data will be addressed similarly. If individual level data are used, we will use standard multiple imputation methods to handle missing data.

### Objective 2: implementation of intervention

#### Outcomes

The protocol is designed to examine not only the direct effects of the intervention but also implementation outcomes, in terms of sustainability, quality of delivery and potential for broader application. Data collection, analysis and integration for implementation outcomes will be guided by the RE-AIM QuEST framework,^21^ which includes quantitative assessments of implementation outcomes, with qualitative assessments to explore the reasons behind quantitative findings.

The mixed-methods approach allows a comprehensive assessment of the intervention’s implementation across five dimensions: Reach, Effectiveness, Adoption, Implementation and Maintenance. Specific outcomes for each of the RE-AIM QuEST framework dimensions are described in table 2, including the sources of quantitative and/or qualitative data for each outcome.

**Table 2:** RE-AIM-QuEST framework applied to 912Rwanda evaluation

Domain	Description	Quantitative measures	Qualitative measures
**Reach**	Engagement with target population, including coverage and representativeness	The use of the intervention including: Crew phone usage (number of crews taking out phones each shift/number of shifts) Phones in use (number) Log-in rate (actual average number of users logged in each day over expected average number) Call volume (calls per day, including location, caller type, condition)	Focus group discussions with dispatch, ambulance crew and facility staff will explore: Reasons for using or not using the 912Rwanda, with attention to influences of organisational context which might support or constrain use How well the intervention reaches target populations Representativeness of target populations
**Effectiveness**	Impact on key outcomes (health, behavioural) and any unintended consequences	Effectiveness outcomes are described in table 1	Focus group discussions with dispatch, ambulance crew and facility staff will explore: Whether users think the intervention is effective and their reasons for thinking whether it is effective or not User perspectives on unintended consequences (positive and negative)
**Adoption**	Extent to which organisations or settings are willing to adopt the intervention	Adoption will be captured as the ‘Criteria for Success’ that would lead to the intervention being rolled out throughout Rwanda. This will be determined through consensus at MCDA workshops (online supplemental file 2). The proportion and representativeness of settings/staff that adopt the intervention.	In-depth interviews with policy makers from MDCA workshops about: The willingness of involved institutions and stakeholders to implement the intervention Whether institutions agree to deliver the intervention and the extent to which they wish to integrate it into existing practices Organisational readiness (including, eg, staffing, infrastructure, resource and/or governance challenges) Factors that could influence adoption such as organisational culture, leadership support and resource availability
**Implementation**	Fidelity, consistency and adaptations in delivery	Implementation will be assessed using data from surveys on Acceptability, Feasibility and Appropriateness.^23^ Fidelity will be assessed using: Proportion of patient journeys where all data for an interface have been completed by users Proportion of patient journeys where any data have been completed for an interface (interfaces include ambulance, dispatch and facilities) Number of times there were user-related hardware failure, user-related software failure, inappropriate use of hardware, internet failure or lack of mobile data Acceptability will be summarised by Number of DDSA decisions overridden by staff (ambulance crew, facility and dispatch) AIM survey Appropriateness will be summarised using IAM survey	In-depth interviews or focus group discussions with dispatch, ambulance crew and facility staff to understand the barriers and facilitators to successful implementation of the intervention regarding: Fidelity to the intervention protocol Quality of delivery of the intervention Acceptability of the intervention Appropriateness of the intervention Reasons given by dispatch for overriding the DDSA Reasons for not fully using the intervention Additionally, a list of adaptions of the intervention will be captured when they are made; these will be summarised, including detail of adaption made, date and reason.
**Maintenance**	Extent to which effects are sustained over time at individual/organisational levels.	Maintenance will be assessed as the continued use of the system, measured by quantitatively drawing on implementation Fidelity outcomes in each interface, 1 year after study end. The proportion and representatives of settings/staff that continue to deliver the intervention of time.	In-depth interviews with policy makers to capture sustainability beyond the duration of the project and possibility of abandonment of intervention, including to which extent the intervention has been integrated into routine practice and continues to be used over time and to explore factors influencing maintenance such as ongoing support, funding and changes in the environment.
**QuEST**			
**Domain**	Description		**Methods**
**Quality**	Consistency and standards of delivery, particularly in terms of delivery and participant experience		In-depth interviews or focus group discussions with dispatch, ambulance crew, facility staff and/or policy makers
**Effectiveness (QuEST**)	Effectiveness re-evaluated with emphasis on real-world conditions, assessing outcomes in varied contexts		
**Scalability**	Potential to expand across larger populations or different settings		
**Transferability**	Adaptability to fit different cultural, organisational or resource contexts		

* Stakeholders include practitioners/health workers/technical implementers/civil servants; decision makers; Ministry of Health officials; political actors; Civil Society Organisations /Non-Governmental Organisations/Principal Investigators/Chief Investgators.

AIM, Acceptability of Intervention Measure; DDSA, destination decision support algorithm; MCDA, Multiple-Criteria Decision Analysis; QuEST, Qualitative Evaluation for Systematic Translation; RE-AIM, Reach, Effectiveness, Adoption, Implementation, Maintenance.

Maintenance will be assessed using quantitative fidelity outcomes collected from 912Rwanda platform in month 12 after the study end. Further funding will be applied to assess this outcome.

In addition, to specifically assess intent to adopt, a Multi-Criteria Decision Analysis (MCDA) workshop will be held to understand additional outcomes which are not captured in the above-described study outcomes which are important to policy makers. Data to inform these additional outcomes will be collected by the study team, as feasible.

#### Data collection

Quantitative implementation data will be collected from 912Rwanda databases and validated surveys conducted with different user groups (ambulance, dispatch and facility staff).

Qualitative data will be collected through focus group discussions with user groups and policy makers. Structured interview and focus group discussion topic guides will be developed based on findings from the quantitative RE-AIM findings and the experiences of the study team during implementation of the intervention. Tailored topic guides will be created to elicit the specific perspectives of each participant group (service users, providers and decision makers).

#### Sample size calculation

No formal sample size calculations will be performed for implementation outcomes, as no hypothesis testing is planned. The sample size for the quantitative outcomes of the RE-AIM framework will be as for the main effectiveness outcome apart from RE-AIM domains which use surveys (in which case, we will attempt to perform surveys with all software users). Qualitative assessment of implementation outcomes will be guided by the objectives of the sub-study, with participants purposively selected to ensure representation of distinct perspectives across two implementation settings, roles and participant groups. Sampling will also be pragmatic, based on practical considerations and resource limitations and thematic saturation.

For the MCDA workshops, we will aim to include 20–25 participants, based on our previous experience indicating that this range allows for diverse perspectives while maintaining operational manageability, discursive coherence and equal participation. This approach ensures a balanced representation of participant insights without compromising the depth and quality of discussions.

#### Data analysis

Quantitative data will be summarised descriptively. For categorical variables, frequencies and percentages will be reported. For numerical variables, means and SD or medians and IQRs will be reported.

Qualitative data will undergo thematic analysis,^22^ with each dataset analysed separately. The analysis will proceed using both inductive coding to identify key emerging themes and deductive coding to align findings with the five RE-AIM QuEST framework domains.

### Objective 3: cost-consequence of intervention

#### Outcomes

A comparative CCA will be performed to ascertain the cost-effectiveness of 912Rwanda compared with usual practice. This will use a health sector approach for costs. This disaggregated approach allows decision makers to determine which consequences are most relevant to their decision-making setting. Resource use and their associated costs for the health sector will be determined. We will also consider the intervention costs (see table 3). We will report resources and costs to enable other countries who are considering using this intervention to calculate local costs and cost savings, giving an ability for more accurate local estimations of investments required, and hence transferability.

**Table 3:** Likely costs and resources included in the cost-consequence analysis

Cost type	Description
Costs and resources used by the health services	Days of stay (eg, bed costs per day)
	Medical investigations
	Medical treatments
	Surgery treatments
	Days of admission to ICU costs per day of ICU stay
	Rehabilitation costs (eg, physiotherapy, occupational therapy, prostheses)
	Costs of transfers to other facilities
Intervention costs and resources (for further roll-out)	*Fixed costs* RWBuild development (eg,staff, testing, equipment and overheads)
	*Variable costs*
	RWBuild maintenance, dependent on the number of users/extent of rollout (eg, staff, equipment, costs incurred in maintaining software and responding to requests from SAMU/RBC/MoH)
	Data collection and input costs at facilities (staff time)
	Report generation costs (staff time for manual data input)

ICU, intensive care unit; MoH, Ministry of Health; RBC, Rwanda Biomedical Centre; SAMU, Service d’Aide Médicale d’Urgence.

#### Data collection

Data to support the CCA will be collected from hospital records/databases, the trauma registry, and supplemented by insurance databases if necessary. Resources associated with the development and operating costs of 912Rwanda have been recorded and will be attributed to the eligible population of those who have and those who could have used the intervention to assign a cost per person for the intervention.

#### Sample size calculation

No formal sample size calculation has been performed.

#### Data analysis

Total costs for pre-intervention and post-intervention will be calculated, accounting for missing data and employing an interrupted time series approach as used in the effectiveness analysis. Mean costs per patient will be compared with the range of consequences estimates by the effectiveness analysis. The impact of explanatory variables (listed in *Objective 1, Explanatory variables*) on costs and consequences will be explored. The scale of cost saving observed will be considered in the context of a potential roll-out across Rwanda, that is, the marginal cost of the intervention (fixed costs) will eventually asymptote to zero. Economic outcomes will be described for both sites combined and separately.

## Discussion

### Overview

This study protocol outlines a comprehensive approach to evaluate the effectiveness, implementation and cost-effectiveness of an electronic DDSA within the 912Rwanda platform, which is designed to improve prehospital transport of injured people in Rwanda. By combining an interrupted time series analysis, the RE-AIM QuEST framework and CCA, we aim to perform a comprehensive, interdisciplinary assessment of the intervention’s effectiveness, implementation process and economic implications.

### Differences in regions

The study will be conducted in Kigali city and Musanze district, representing predominantly urban and rural settings, respectively. Kigali, the capital and largest city of Rwanda, is in the centre of the country, while Musanze is situated in the Northern Province of Rwanda. In both regions, the public ambulance service, SAMU, carries emergency patients to all public and private health facilities. This approach allows us to understand how the intervention’s effectiveness may vary across urban and rural settings.

### Strengths and limitations

The study has several strengths, including the use of interrupted time series, which provides a robust quasi-experimental approach for evaluating intervention’s effectiveness when randomisation is not feasible. The implementation of the intervention is assessed using the RE-AIM QuEST framework which allows for comprehensive understanding through both quantitative and qualitative outcomes. Additionally, the CCA will enable decision-makers to weigh the trade-offs between different costs and outcomes. However, the study also has limitations. The generalisability of findings may be challenging due to variations in contexts and resource availability across countries, potentially limiting the applicability of findings. The data collection method is different for the baseline period compared with phase one and phase two of the intervention, and different across the locations, which may lead to inconsistencies in the data. In Kigali, data are primarily collected through the TDCA app or the Rwanda912 platform, with paper forms used as a backup if electronic data capture fails, on which data quality checks will be performed. In contrast, in Musanze, data for the pre-intervention phase is collected solely on paper and inputted electronically, which presents a limitation in terms of data reliability for that phase. Furthermore, the rapidly evolving nature of software development and technologies may lead to research findings becoming outdated quickly, making it essential to stay updated with the latest developments.

### Ethics and dissemination

Ethical approval has been obtained from the Rwanda National Research Ethics Committee (Ref No: 99/RNEC/2023). Findings from the study will be disseminated through open access peer-reviewed publications, and relevant national and international conferences.

## Supplementary material

10.1136/bmjopen-2025-100826 online supplemental file 1
