# Supplementary material for: Use of an innovative electronic communications platform (912Rwanda) to improve prehospital transport of injured people in Rwanda: protocol for a type 2 hybrid effectiveness-implementation interrupted time series study
Source: BMJ Open. 2025 Aug 13;15(8):e100826. doi: 10.1136/bmjopen-2025-100826 (PMC12352135; doi:10.1136/bmjopen-2025-100826)
Supplement: online supplemental file 1 [file bmjopen-15-8-s001.docx]

**Supplementary Material 1.** Proportion of emergency patients received by hospitals in Kigali from SAMU.

| **Hospital** | **Percentage** |
| --- | --- |
| Kibagabaga District Hospital | 21% |
| Nyarugenge District Hospital | 16% |
| Centre Hospitalier Universitaire de Kigali | 13% |
| Muhima District Hospital | 8% |
| Masaka District Hospital | 5% |
| King Faisal Hospital | 3% |
| Rwanda Military Hospital | 3% |
| Kacyiru District Hospital | 1% |

Note: The remaining 31% are mainly transferred to health centres or managed on site (minor cases).

**Supplementary Material 2. MDCA workshops**

**MDCA plan**

MCDA will be conducted through facilitated discussion sessions with policy makers at the start and the end of the study. Discussion sessions will be led by Laura Bojke (Co-I) who has experience of development of preference elicitation and Lucia D’Ambruoso, the lead qualitative researcher with expertise in translation of evidence to policy in LMICs.

MCDA proceedings will follow this ISPOR taskforce report; it will consist of 5 steps:

1. Define the decision problem (start of the study)
2. Identification of criteria and building consensus (start of the study)
3. Build a performance matrix (see example below) to illustrate the criteria and trade-offs; this will incorporate quantitative and qualitative evidence from the study (end of study)
4. Weighting of criteria according to preferences (end of study)
5. Feedback and consideration (end of study)

**Example of a decision matrix for the MCDA.**

|  | **Implementation cost** | **Mean response time** | **Mean survival** | **Equity impact** |
| --- | --- | --- | --- | --- |
| Rwanda912 | $25 p.p | 25 mins | 25 months | Yes |
| Standard care | $0 | 35 mins | 20 months | No |

Note: Some of these criteria are measured in cardinal numbers, some in binary terms and others can be measured in qualitative terms

At the end of the study, given the criteria and observed differences and their associated uncertainties, decision makers will be able to determine if criteria for success have been met according to the weighting of alternatives, i.e. a change in response time has been observed but an increase in costs observed (compensatory technique).

**Participants**

20-30 participants will attend each workshop

*Participants (Rwanda)*

Local participants to the MCDA facilitated discussions will be:

- SAMU leadership
- RBC leadership
- MoH

*Participants (elsewhere)*

At the start of the study, we will run a Policy Workshop using MCDA methods with purposively selected stakeholders from other LMICs to capture their criteria for success, focussing on steps 1 and 2 of the MCDA process; this will enable us to outline additional data to capture in this study to facilitate rollout in other countries.

**Proceedings**

A 1/2-day workshop will be held with all participants at the start and at the end of the study (Rwanda only). The workshop at the end of the study will be delivered once analyses have been done from both Kigali and Musanze (between March and May 2027)

Sessions will be in plenary and round table. We are likely to use a nominal group or modified Delphi process as we will be seeking to reach consensus on criteria See further details in next section for the first and second stage MCDA process.

**Data capture**

*MCDA outputs*

Data will be the consensus outputs from discussions. These will be summarised, and any areas of disagreement described. It may be possible to generate relative weights/rankings for each of the criteria, i.e. describe the order of importance for criteria to be met. Discussions will also be recorded in case of need for later clarity when preparing the report.

*Qualitative interviews*

In-depth interviews will be done with policy makers who have expressed compelling opinions during the second stage MCDA process to assess their experiences with the intervention or their reasons for delineating facilitators or barriers to roll out.

**Plans for roll out**

After completion of the MCDA exercise. If it has been agreed that sustainment and roll out is appropriate, there will be a facilitated workshop discussion on the plans for sustainment and roll out, the hurdles that may present and suggestions to overcome these. See next sections for further details appendix 9.

**Data use**

A report will be prepared which describes the process and the criteria and thresholds for success. This will be used for later discussions of whether to roll out the intervention more widely or not. Any criteria or thresholds that are not being captured in the data collection and which are feasible to collect will be added to the data collection described above. After the initial MCDA workshops, a provisional matrix will be created by the research team to display the criteria/thresholds and potential trade-offs. This will be completed prior to the final MCDA workshop by the research team using results from the study. The results of the final MCDA workshop will be written up by the research team to show whether criteria for success – as defined in the first workshop – have been met, what plans for roll out are, and any expected hurdles.

**MCDA workshop proceedings**

We will follow the ISPOR Good Practice Guidelines for MCDA to conduct the before and after MCDA sessions. Observers will capture listed outcomes from the meeting and also note any points of discussion and disagreement. They will additionally capture the names of any contributors who have expressed compelling or contradictory opinions during the workshop and note what those opinions were about; they will approach these participants and request a further 30 minute interview to explore these further.

*First stage MCDA workshop with local policy makers and international policy makers (stakeholders)*

1. Welcome and introductions
2. Presentation to introduce the MCDA process and expectation of stakeholders
3. Participants divide into small roundtable groups to discuss the decision problem and provide some initial thoughts on metrics to measure success
4. Participants present the results of their small group discussions in plenary with opportunities for the floor to ask questions on reasons behind the problem identified and metrics.
5. Consensus on definition and metrics are agreed in facilitated plenary
6. Roundtable discussions on changes in metrics (change criteria) with which to judge whether the solution to the decision problem has been successful
7. Plenary presentations of roundtable discussions on change criteria with opportunities for the floor to ask questions
8. Consensus on change criteria agreed in plenary discussion or by further roundtable discussions if necessary

*Second stage MCDA workshop (at end of intervention) with local policy makers*

*Part 1*

1. Welcome and introductions
2. Introduction to the process in plenary, expectations of stakeholders and recap of agreed metrics and change criteria from first workshop
3. Presentation of the study results and the completed MCDA criteria/trade off matrix
4. Participants divide into small roundtable groups to discuss their prioritisation and their rationale for this prioritisation
5. Participants present the results of their small group discussions in plenary with opportunities for the floor to ask questions on reasons behind their prioritisation.
6. Consensus on prioritsation are agreed in plenary and if consensus is not obtained, there will be a further round of roundtable discussions /plenary
7. Roundtable discussions on whether sufficient criteria have been met to roll-out the intervention in Rwanda and the reasons behind the decision.
8. Plenary presentations of roundtable discussions on change criteria with opportunities for the floor to ask questions

*In depth interview guide for policy makers who have expressed compelling opinions during the MCDA workshop*

Thank-you for taking part in the workshop to determine criteria that you think is useful to assess whether the communication tool is useful. Some of the points you discussed were interesting to us for the following reasons and we would like to ask you some questions about those.

Questions will be developed by the research team and specific to the view expressed by the participant.

**Plans for roll out**

After consensus has been developed during the MCDA workshop on whether criteria for roll out have been met, a facilitated discussion will capture plans for roll out, any anticipated hurdles, and plans to overcome these hurdles. The discussion will proceed as follows:

1. Roundtable discussion on plans for roll-out – focusing on how this would be delivered
2. Plenary discussion with groups presenting their roundtable conclusions with further discussion until consensus on how roll out would occur is agreed
3. Roundtable discussion on anticipated hurdles to roll-out and how these would be overcome
4. A plenary discussion with groups presenting their roundtable conclusions and further discussion and consensus on whether hurdles are important and how to overcome them
