# Supplementary material for: Use of an innovative electronic communications platform (912Rwanda) to improve prehospital transport of injured people in Rwanda: protocol for a type 2 hybrid effectiveness-implementation interrupted time series study
Source: BMJ Open. 2025 Aug 13;15(8):e100826. doi: 10.1136/bmjopen-2025-100826 (PMC12352135; doi:10.1136/bmjopen-2025-100826)
Supplement: Supplementary file 2 [file bmjopen-2025-100826.reviewer_comments.pdf]

PEER REVIEW HISTORY

BMJ Open publishes all reviews undertaken for accepted manuscripts. Reviewers are asked to complete a checklist review form and are provided with free text boxes to elaborate on their assessment. These free text comments are reproduced below.

ARTICLE DETAILS

Title (Provisional)

Use of an innovative electronic communications platform (912Rwanda) to improve pre-hospital transport of injured people in Rwanda: protocol for a type 2 hybrid effectiveness-implementation interrupted time series study

Authors

Group, Rwanda912 RIGHT

VERSION 1 - REVIEW

**Reviewer**	**1**
**Name**	**Dale, Megan**
**Affiliation**	**Cardiff and Vale University Health Board**
**Date**	**12-Mar-2025**
**COI**	**None**

The study is very interesting,and the protocol is clearly described and well written.

Data collection on paper is discussed briefly in the limitations, but I imagine it could be very challenging for items such as times for events. It would have been interesting to hear a little more about how this will be overcome.

**Reviewer**	**2**
**Name**	**McCartin, Michael**
**Affiliation**	**The University of Chicago Medicine**
**Date**	**26-May-2025**
**COI**	**None**

Thank you for the opportunity to review this manuscript. It is well-written and well-structured. Reducing transport times is essential for many life threatening disease processes (trauma, STEMI, stroke) - so anything that might make an impact on expedient transport to an appropriate destination with definitive care is well worth pursuing. I only have a few very minor suggestions to help clarify key points:

1. “Initial explorations found that inefficient mobile phone communication and coordination between ambulance, dispatch, and receiving hospitals, and lack of efficient usage of triage data from ambulance staff resulted in an average time to reach hospital of around one hour.”

That’s accurate, but is a little abstract. For readers who don’t work in EMS, you might consider spelling out why this poor communication causes delay. It could help non-EMS readers better understand the real-world implications..

For instance: “One major consequence of this poor communication is that ambulance crews often lack enough information to find their patients quickly—wasting time that could be used transporting patients to care.”

2. Similarly to above – Figure 1 is helpful, but the caption could be expanded to really highlight what’s changing between Part A and Part B of the comms figure. This might enable to the figure to stand on its own more easily – and not require the background of the article to have been read.

3. Since the primary outcome is based on time intervals between dispatch and hospital arrival, it would be useful to clarify whether time stamps are generated from synchronized systems. For example, if dispatch and EMS are using different clocks—or if some times are manually entered—this could introduce noise or bias.

4. Given that one of the main problems this intervention seeks to address is fragmented communication between dispatch, EMS crews, and receiving hospitals, it’s reasonable to assume that pre-intervention time data may be inconsistently recorded or incomplete. It may be helpful to briefly acknowledge this limitation and note whether any steps will be taken to audit or validate early data (even via sampling).

VERSION 1 - AUTHOR RESPONSE

**Reviewer: 1**<br>**Dr. Megan Dale, Cardiff and Vale University Health Board**		
The study is very interesting,and the protocol is clearly described and well written.<br>Data collection on paper is discussed briefly in the limitations, but I imagine it could be very challenging for items such as times for events. It would have been interesting to hear a little more about how this will be overcome.	Thank you for your comment. We have updated the limitations section to clarify that in Kigali, the paper forms serve as a backup and will undergo data quality checks if it is needed. For the pre-intervention period in Musanze, data is only collected by paper, and this limitation has been clarified.	pg21<br>The data collection method is different for the baseline period compared to phase one and phase two of the intervention, and different across the locations, which may lead to inconsistencies in the data. In Kigali, data is primarily collected through the TDCA app or the Rwanda912 platform, with paper forms used as a backup if electronic data

		capture fails, on which data quality checks will be performed. In contrast, in Musanze, data for the pre-intervention phase is collected solely on paper and inputted electronically, which presents a limitation in terms of data reliability for that phase.
**Reviewer: 2**<br>**Dr. Michael McCartin, The University of Chicago Medicine**		
Thank you for the opportunity to review this manuscript. It is well-written and well-structured.<br>Reducing transport times is essential for many life threatening disease processes (trauma, STEMI, stroke) - so anything that might make an impact on expedient transport to an appropriate destination with definitive care is well worth pursuing.<br>I only have a few very minor suggestions to help clarify key points:	Thank you for your comment.	
1. "Initial explorations found that inefficient mobile phone communication and coordination between ambulance, dispatch, and receiving hospitals, and lack of efficient usage of triage data from ambulance staff resulted in an average time to reach hospital of around one hour."<br>That's accurate, but is a little abstract. For readers who don't work in EMS, you might consider spelling out why this poor communication causes delay. It could help non-EMS readers better understand the real-world implications..<br>For instance: "One major consequence of this poor communication is that ambulance crews often lack enough information to find their patients quickly—wasting time that could be used transporting patients to care."	Thank you for your suggestion. We have revised the introduction section to clarify the consequences of poor communication.	pg5<br>One major consequence of this poor communication is that ambulance crews often lack enough information to find their patients quickly, to identify an appropriate receiving hospital for the patient, or to prepare the receiving hospital in advance, wasting valuable time that could be used transporting patients to care.
2. Similarly to above – Figure 1 is helpful, but the caption could be expanded to really highlight what's changing between Part A and Part B	Thank you. We agree and have now revised the caption for Figure 1 to	pg25<br>Figure 1. The top figure (Part a) represents the current inefficient

of the comms figure. This might enable to the figure to stand on its own more easily – and not require the background of the article to have been read.	explain the differences between Part A and Part B.	communication system, which relies on separate mobile phone and Very High Frequency (VHF) radio communication between the dispatch centre, ambulance crews, and health facilities, resulting in pre-hospital delays. The bottom figure (Part b) represents the proposed 912Rwanda platform, an integrated electronic system designed to connect dispatch, ambulance crews, and health facilities to reduce pre-hospital delays.
3. Since the primary outcome is based on time intervals between dispatch and hospital arrival, it would be useful to clarify whether time stamps are generated from synchronized systems. For example, if dispatch and EMS are using different clocks—or if some times are manually entered—this could introduce noise or bias.	Thank you for the comment. We have now clarified in the methods section about the how the time data is collected and that is through a synchronised system.	pg10<br>All electronic time stamps (TDCA and 912Rwanda) are generated automatically from synchronized system clocks to ensure consistency; manual entry of time data is only used as a backup.
4. Given that one of the main problems this intervention seeks to address is fragmented communication between dispatch, EMS crews, and receiving hospitals, it’s reasonable to assume that pre-intervention time data may be inconsistently recorded or incomplete. It may be helpful to briefly acknowledge this limitation and note whether any steps will be taken to audit or validate early data (even via sampling).	Thank you for your comment. We acknowledge that pre-intervention data may be inconsistent or incomplete, however, we rolled out an electronic time data collection app prior to the intervention to increase precision of the time data. In Kigali, we also have the back-up time collected data on paper if needed to cross check electronic data quality. In Musanze where the pre-intervention data is only available on paper records, this limitation has been acknowledged.	pg21<br>The data collection method is different for the baseline period compared to phase one and phase two of the intervention, and different across the locations, which may lead to inconsistencies in the data. In Kigali, data is primarily collected through the TDCA app or the Rwanda912 platform, with paper forms used as a backup if electronic data capture fails, on which data quality checks will be performed. In contrast, in Musanze, data for the pre-intervention phase is collected solely on paper and inputted electronically, which presents a limitation

		in terms of data reliability for that phase.
